# Analytic Sensitivity of an ELISA Test on Pooled Sera Samples for Detection of Bovine Brucellosis in Eradication Stages in Uruguay

**DOI:** 10.3389/fvets.2020.00178

**Published:** 2020-04-15

**Authors:** Joaquin Baruch, Alejandra Suanes, Jose M. Piaggio, Andres D. Gil

**Affiliations:** ^1^Departamento de Bioestadística, Facultad de Veterinaria, Universidad de la Republica, Montevideo, Uruguay; ^2^Center for Outcomes Research and Epidemiology, College of Veterinary Medicine, Kansas State University, Manhattan, KS, United States; ^3^Departmento de Bacteriología, Dirección de Laboratorios Veterinarios, Ministerio de Ganadería, Agricultura y Pesca, Montevideo, Uruguay

**Keywords:** Brucellosis, diagnostics, surveillance, Uruguay, eradication

## Abstract

Bovine brucellosis has been under eradication in Uruguay since 1998. The eradication program includes, among other interventions, individual sera sampling of beef animals at slaughter, and annual serum testing of all dairy cows—accounting for two million samples annually. At a herd prevalence of 0.8%, a pooled-sera sample approach could reduce the economic burden of the surveillance system by reducing the testing and operational costs. Our objective was to evaluate the analytic sensitivity of an indirect ELISA test for *Brucella abortus* in serum pools. Sixty-two *Brucella abortus-*positive bovine sera samples (based upon rose bengal and fluorescent polarization assay) were used as the positive control samples. Rose bengal-negative sera from negative farms were used to dilute the positive samples to the desired concentrations. Positive samples were diluted by using 1 ml of positive sera and 1 ml of negative sera (1/2 dilution) up to 1/1,024. Data were analyzed using generalized linear mixed models with a binary outcome (positive or negative), dilution number as a fixed effect, and a random effect for sample ID. Analytic sensitivity was 99.0% [95% confidence interval (CI): 96.3–99.7], 98.3% (95% CI: 93.1–99.6), 97.3% (95% CI: 87.4–99.4) for dilutions 1/2, 1/4, and 1/8, respectively. The analytical sensitivity, however, decreased when diluted to greater proportions. Given the current herd prevalence in Uruguay, it seems plausible that the use of a pooled sample approach could be adopted by policymakers to reduce the cost of the surveillance program and increase the number of samples being tested.

## Introduction

Bovine brucellosis is a worldwide-distributed zoonosis that causes abortions in cattle and undulant fever in humans. It has been reported that *Brucella spp*. causes the largest number of zoonotic infections worldwide, with more than 500,000 new cases in humans each year (1). Bovine brucellosis is present in all countries across Latin America with Mexico and Peru having a relatively high prevalence; however, Uruguay has relatively low prevalence estimated to be 0.2% at an animal-level and 0.8% at a herd-level (2–5). Abortions lead to a decreased number of calves per year, and reduced milk production, having economic impacts on beef and dairy producers. According to McDermott et al. (6), in high-income countries, cows that had a bovine brucellosis abortion will have an economic loss of 20–25% during that season. In Uruguay, Piaggio et al. (4) estimated that the cost of sanitary measures applied to outbreak control every year (i.e., bleeding, vaccination, diagnosis, and indemnity) is on average $US 2,360,000. The general pattern of eradication is based on diagnostic testing and culling of positive cattle. However, during the initial stages of eradication, vaccination is usually implemented (4). As in any disease eradication process, the final steps have a high cost per positive diagnostic and it is necessary to use tests that are accurate at a low price.

Bovine brucellosis has been under an eradication campaign in Uruguay since 1998. Currently, surveillance activities are carried out by government veterinarians with screening samples being collected by private veterinarians with government accreditation. All dairy cattle are tested annually by a screening Rose Bengal (RB) test, and in case of a positive test, a government laboratory performs a second test in series with fluorescent polarization assay. Moreover, an indirect ELISA test in bulk milk is conducted every 4 months. For beef farms, testing of animals occurs when animals go to slaughter, in which the intention is not to detect individual animals but to detect suspected farms. Cattle going to exhibitions, trade fairs, and for exports are tested before movement, while those coming from areas declared as endemic by the government must have RB-negative serology before movement (7). Given the extensive surveillance system, there are about 2,000,000 samples screened for bovine brucellosis annually (8). Bovine brucellosis is in an advanced stage of eradication in Uruguay. The World Organization for Animal Health (OIE) states that a country or region free of bovine brucellosis is one that has <0.2% of herds with the presence of the disease; no vaccinated animals in the last 3 years; and any positive animal has been slaughtered.

It is assumed that the RB test has a sensitivity of 97.7% and a specificity of 99.9% (9). However, there are reports in the literature of much lower sensitivities (Table 1). Table 1 depicts the sensitivity and specificity of the tests that are used in Uruguay. The indirect ELISA test has a high sensitivity for bovine brucellosis (Table 1). Using this advantage, we hypothesized that pooled samples could be used to reduce testing costs and increase the number of samples evaluated per unit of time. In the event a pooled test is positive, identifying the animal or animals that gave the positivity to the pool should be determined by testing samples individually. Given the low prevalence of the disease, most pooled tests will be negative, which might significantly lower the costs of the eradication campaign. Therefore, our objective was to evaluate the analytical sensitivity of an indirect ELISA test in pooled sera samples as a tool for epidemiological surveillance of bovine brucellosis in Uruguay.

**Table 1:** Summary of sensitivity and specificity of the current tests used on the national surveillance of bovine brucellosis in Uruguay.

**Test**	**Se^**a**^**	**95% CI^**b**^**	**Sp^**c**^**	**95% CI^**b**^**	**Reference**
Rose bengal	97.7	95.9–99.3	99.9	99.8–99.9	(9)
	89.6	79.9–95.8	84.5	68.0–94.8	(10)
Competitive ELISA	98.4	97.0–99.8	99.4	99.1–99.6	(9)
Indirect ELISA	95.7	93.4–98.0	99.8	99.7–99.9	(9)
	96.8	92.3–99.1	96.3	91.7–98.8	(10)
Complement fixation	94.0	87.8–97.5	88.5	81.0–93.8	(10)
Fluorescent polarization assay	96.4	94.4–98.5	99.9	99.7–99.9	(9)

a *Confidence interval*.

b *Sensitivity percentage*.

c *Specificity percentage*.

## Materials and Methods

This study did not require approval by the Honorary Commission of Animal Research of the Faculty of Veterinary Medicine, University of the Republic, due to having no involvement with animal subjects. All samples were obtained from a national sera bank provided by the Ministry of Agriculture.

### Samples and Laboratory Procedures

Sixty-two bovine brucellosis positive sera were obtained from a sera bank. Positive samples were defined as (1) sera that had tested positive to both RB and fluorescent polarization assay; and (2) originated from a positive farm. Positive farms are defined by the Ministry of Agriculture based on an epidemiological investigation that takes into account the number of positive animals, animal movements, vaccination history, prior serology, abortion history, and location (11). Negative sera were obtained from farms that had no history of bovine brucellosis in the previous 5 years and were negative to RB.

Positive samples were diluted by adding 1 ml of positive sera to 1 ml of negative sera (1/2 dilution), with subsequent dilutions performed by adding 1 ml of the previous dilution to 1 ml of negative sera and homogenizing (up to 1/1,024 dilution). All 62 positive samples and the dilutions were analyzed by an indirect ELISA test of the Pourquier Institute (IDEXX Laboratories, Westbrook, ME); therefore, a total of 682 samples were analyzed. Dilutions were diluted in 1/20 using 10 μl of the sample and 190 μl of the kit's diluent; the same dilutions were performed for the negative and positive controls provided by the kit. Sample dilutions were placed on the plate, covered, and incubated for 1 h at room temperature. After that, the washing solution was diluted in 1/20 using distilled water, the plate's contents were emptied, and the plates were washed three times. The conjugate was diluted in a 1/100 dilution using the kit's buffer “number 1,” and 100 μl were placed on each well. Plates were incubated for 1 h at room temperature covering the plate on its aluminum foil. After the incubation period, the washing step was repeated, and 100 μl of the revelation solution were placed in each well. Plates were incubated for 20 min and 100 μl of the stop solution were dispensed into each well. Optical densities of 450 nm were used for reading the plates on a conventional ELISA reading instrument. Plates were considered valid if the average of the optical density of both positive controls was >0.6, and the ratio of the average of the optical density of both positive controls over the negative control was >2. The sample to positive (S/P) ratio was calculated using Equation 1 where OD450 is the optical density reported by the ELISA instrument at an optical density of 450.

$$\begin{array}{r} \frac{S}{P}\%=100\;X\frac{OD450\;value\;of\;the\;sample-OD450\;value\;of\;the\;negative\;control}{mean\;OD450\;value\;of\;the\;positive\;control-OD450\;value\;of\;the\;negative\;control} \end{array}$$

A positive result was assigned to the dilution if the S/P% was >120. Two operators (a veterinarian and a veterinary student) implemented all laboratory procedures and were not blinded to the true status of the samples. Despite the previous laboratory experience of the operators, the Ministry of Agriculture provided training on laboratory techniques and biosecurity measures. All procedures were performed at the Faculty of Veterinary Medicine of the University of the Republic, Montevideo, Uruguay. After processing, samples were immediately frozen in cryogenic vials for further investigations with other tests.

### Statistical Analysis

A generalized linear mixed model was fitted to estimate the analytical sensitivity of the ELISA test on different dilutions using the GLIMMIX procedure in SAS 9.4 (SAS Institute Inc., Cary, NC). The ELISA test result (positive vs. negative) was modeled as the outcome with a binomial distribution, logit link, and a residual pseudo-likelihood estimation technique. The natural logarithm (ln) of the dilution number was modeled as a continuous fixed effect, and a random effect for sample ID, with a first-order autoregressive correlation structure, was included to account for the repeated measures data structure. The dilution number was modeled on the ln scale to meet the linearity assumption. Analytical sensitivity estimates and confidence intervals were obtained using Equation 2.

$$\begin{array}{r} \text{Analytical sensitivity }(\text{dilution X})=\frac{e^{\left(ln(dilution\;X)*\beta (dilution)+\beta (intercept)\right)}}{e^{\left(\mathrm{In}(dilution\;X)*\beta (dilution)+\beta (intercept)\right)}+1} \end{array}$$

Where *analytical sensitivity (dilution X)* is the analytical sensitivity for a given dilution, *ln(dilution X)* is the natural logarithm of dilution X, β*(dilution)* is the model coefficient for dilution number, and β*(intercept)* is the model coefficient for the model intercept.

## Results

**Figure 1:**
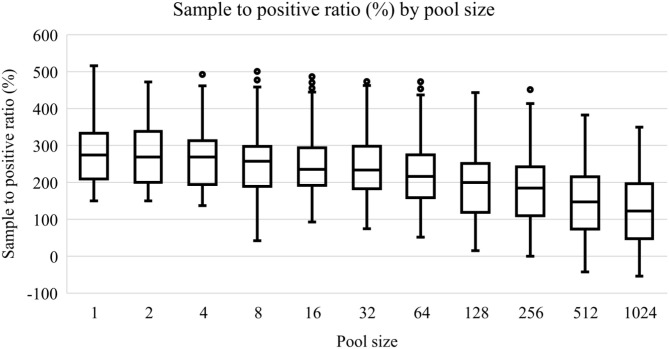
Box-plots of sample to positive ratios (%) of an ELISA test to detect bovine brucellosis antibodies in pooled sera samples. A positive pool was determined if the sample to positive ratio was greater than 120.

All initial 62 positive samples were included in the study and tested positive by the indirect ELISA test. The average OD450 of the 62 positive samples was 2.11 (standard deviation = 0.69, range = 0.25–3.83) and Figure 1 depicts the S/P% of each dilution. Table 2 depicts the number of positives and negatives in each dilution. Model coefficients and analytical sensitivities for the dilutions used in this study are depicted in Table 3.

**Table 2:** Descriptive results of dilutions of positive sera samples to bovine brucellosis analyzed with an indirect ELISA test.

**Dilution**	**Negative**	**Positive**	**Total**
1	0	62	62
2	0	62	62
4	0	62	62
8	2	60	62
16	2	60	62
32	5	57	62
64	7	55	62
128	15	47	62
256	18	44	62
512	24	38	62
1024	31	31	62

**Table 3:** Model outputs and estimated analytic sensitivities for an indirect ELISA test to detect bovine brucellosis antibodies in pooled samples (dilution).

**Effect**	**Coefficient**	**SE**	**95% CI^**a**^**
Ln (dilution)^*b*^	−0.75	0.10	−0.95 to −0.55
Intercept	5.13	0.61	3.91–6.34
**Dilution**	**Sensitivity (%)**		**95% CI**^**a**^
1	99.4		98.0–99.8
2	99.0		96.3–99.7
4	98.3		93.1–99.6
8	97.3		87.4–99.4
16	95.5		78.3–99.2
32	92.6		65.2–98.8
64	88.2		49.4–98.3
128	81.6		33.6–97.5
256	72.6		20.8–96.4
512	61.1		12.0–94.8
1,024	48.4		6.6–92.5

a *CI, Confidence interval*.

b *Dilution number was transformed to the natural logarithmic scale to meet the linearity assumption*.

## Discussion

Our study suggests that the use of an ELISA test in pooled sera samples may be an appropriate testing method for bovine brucellosis screening in Uruguay. This study sought to determine the analytic sensitivity of a pooled testing method for its implementation in epidemiological surveillance scenarios, which might be a more economical alternative than testing individual samples. Health authorities could use this testing method for low-risk farms or slaughterhouse sampling (in which the goal is to determine groups of positive animals, rather than individuals, in order to identify the farm of origin).

The ELISA kit manufacturer recommends that pools of up to 10 sera can be performed (12). The manufacturer, however, does not provide an estimate of the sensitivity for pooled samples. This study contributes to the scientific community, specifically to the Ministry of Agriculture, in providing an estimate of the sensitivity of pooled samples in the Uruguayan cattle population. Pools of up to 16 samples could be used if the Ministry of Agriculture considers a relative sensitivity close to 95% as acceptable for regions in which, presumably, the disease is not present. If the testing situation requires a higher sensitivity, such as an area with a higher bovine brucellosis risk, pools of 4 samples could be implemented. Similarly, by decreasing the S/P% cutoff, higher test sensitivities could be achieved without reducing the pool size. Lowering the S/P% cutoff, however, would decrease the specificity, which will increase the number of false-positive pools, increasing the number of samples tested individually.

The use of pools for testing diseases is well-established in both human and animal medicine (13). In the case of bovine brucellosis, pooled samples are currently used to monitor milk samples from dairy herds in Uruguay. The use of highly sensitive tests, such as the indirect ELISA test, has replaced the previously used milk ring test and could also replace the RB test if the cost/benefit ratio is favorable (7). There is, however, a need for an economic analysis taking into account the testing costs (kit and labor), pool size, sensitivity, specificity, and prevalence. This type of analysis has been performed in the past and should be conducted before adopting this testing scheme at a national level (14).

The lack of a gold standard method for the positive reference sera and the limited number of samples were among the limitations of this study. The former was addressed, in order to limit information bias, by using two tests to confirm that the sera were bovine brucellosis true-positive samples and by obtaining positive samples from farms that were considered infected. It is expected that the initial samples were true positive given the high specificity of the RB test and the fluorescent polarization assay used. Another limitation of this study was the lack of prior knowledge regarding the animals that were used to obtain the sera samples. Knowledge of the age categories, breed, and region within the country would have been useful to assess possible confounders. Also, lack of farm identification limited the possibility of accounting for the clustering of animals within farms. No negative samples were tested in this study, which could be a limitation of this study if the test specificity is reduced when pooling samples. A reduction in test specify would increase the number of false-positive pools, and therefore, increase the testing cost. To our knowledge, however, there are no studies indicating a reduction in test specificity when performing ELISA tests in pools for bovine brucellosis. Despite the fact that this test has already been validated for pooled samples by the manufacturer, future research should study the interoperator and intraoperator repeatability of this test and the potential dilution effect on the specificity.

In the current situation of a 0.8% herd prevalence of bovine brucellosis in Uruguay, a pooled test may be useful, because the probability that a pool is positive in bovine brucellosis-free farms under surveillance is low. With a surveillance system that analyzes 2,000,000 samples per year, we recommend the use of the pooled test with retesting of individual samples within positive pools. Given the relevance of bovine brucellosis in Uruguay, further research is necessary to ensure there is no dilution effect on the specificity while accounting for the clustering of animals within farms.

## Data Availability Statement

The datasets generated for this study are available on request to the corresponding author.

## Ethics Statement

This study did not require approval by the Honorary Commission of Animal Research of the Faculty of Veterinary Medicine, University of the Republic, due to having no involvement with animal subjects. All samples were obtained from a national sera bank provided by the Ministry of Livestock, Agriculture, and Fisheries.

## Author Contributions

JB wrote the manuscript, helped design the study, and performed the laboratory work and data analyses. AS provided training on laboratory techniques and assisted in study design. JP and AG assisted with the statistical analyses and designed the study. Finally, all co-authors reviewed the manuscript.

### Conflict of Interest

The authors declare that the research was conducted in the absence of any commercial or financial relationships that could be construed as a potential conflict of interest.
